# The burden of COVID-19 pandemic on tuberculosis detection: a single-center study

**DOI:** 10.1186/s43168-022-00117-x

**Published:** 2022-03-18

**Authors:** Maiada K. Hashem, Aliae A. R. Mohamed Hussein, Mariam Taher Amin, Abdelmalek Mahmoud, Ahmad M. Shaddad

**Affiliations:** 1 Chest Department, Faculty of Medicine, Assiut University, Assiut, 71515 Egypt; 2 Public Health and Community Medicine Department, Faculty of Medicine, Assiut University, Assiut, Egypt; 3 Assiut Chest Hospital, Assiut, Egypt

**Keywords:** Tuberculosis, Case detection, Decline, COVID-19, Pandemic, Burden, Pulmonary, Extrapulmonary TB, Low-income countries

## Abstract

**Background:**

Since being declared a global pandemic, Coronavirus disease 2019 (COVID-19) took over healthcare providers and researchers’ interest. However, other epidemic diseases, including tuberculosis (TB), are still a health issue that aggravate under the umbrella of health facilities exhaustion. This study aims to evaluate the impact of the COVID-19 pandemic on tuberculosis management.

**Methods:**

A retrospective analysis of the quarterly reports issued by a tuberculosis management unit from 2017 to June 2021, including data of 12 subunits. The changes in pulmonary and extrapulmonary tuberculosis incidence trends (new + relapsed cases) throughout the 4 years were reported. The quarterly changed percentages in cases numbers along 2020 and first half of 2021 was compared with that of the same periods in 2019.

**Results:**

Incidence of extrapulmonary tuberculosis was higher than pulmonary tuberculosis throughout the 4 year study periods (7.69 vs. 4.49, 9.44 vs. 4.33, 7.75 vs. 3.58, and 7.82 vs. 2.94/100.000 population, respectively) with a noticeable decline in the incidence of pulmonary TB during 2020. The second quarter of 2020 showed the lowest tuberculosis incidence rate with a 41.6% decline in the total number of diagnosed cases while 2nd quarter of 2021 showed 21.2% decline. During 2020, only 4 cases of multidrug-resistant TB were reported (compared to an average of 8 cases of MDR-TB yearly before the COVID-19 era).

**Conclusion:**

There was a noticeable drop in tuberculosis case detection during the COVID-19 pandemic. The lockdown, started in Egypt by the end of March 2020, could contribute to the marked drop in the second quarter. However, a steady partial decline was continued during the first half of 2021, which foretells a growing problem.

## Introduction

Since being declared a global pandemic due to its effect on public health and the economy, coronavirus disease 2019 (COVID-19) became the top interest of healthcare providers, scientists, and researchers. However, other epidemic communicable diseases, including tuberculosis (TB), are still a health problem that may grow and drain health facilities [1]. According to the global tuberculosis report 2020, an estimated 10 million people are diagnosed with tuberculosis, with 1.4 million deaths worldwide [2]. In 2019, the incidence of tuberculosis in Egypt was estimated to be 12 cases per 100,000 people [3].

There is growing evidence on the biological, clinical, and epidemiological interaction between tuberculosis and COVID-19 disease [4]. Moreover, patients suffering from comorbid respiratory conditions have impaired lung function and altered immune defense mechanisms putting them at higher risk of developing more severe COVID-19 infection [5]. A recent meta-analysis indicated that tuberculosis was related to a doubled risk of COVID19 [6]. Unlikely, the response of the COVID-19 pandemic, particularly the transfer of health care equipment and employees and containment measures, affects care initiatives and tuberculosis prevention [7, 8]. It was suggested that a lockdown of 3 months followed by an extended recovery of 10 months might result in 1.4 million tuberculosis deaths and an additional 6.3 million tuberculosis cases from 2020 to 2025. Such statistics would imply a 5–8-year decline in the fight against tuberculosis [9]. However, these early modeling investigations depended on assumptions that should be readdressed considering empirical data. Since conducting these analyses, no systematic information has been gathered to quantify the influence of COVID-19 on the burden of tuberculosis [10].

The first COVID-19-positive case in Egypt was detected on 3 January 2020, and the lockdown was followed, aiming to limit its spread on 24 March 2020 [11]. To our knowledge, no reports consider the impact of the pandemic and its precautionary measures on healthcare services, including the TB national program in Egypt.

This study aims to estimate the effect of the COVID-19 pandemic on tuberculosis case detection in one of the Upper Egypt governorates and evaluate its burden on tuberculosis management services.

## Patients and methods

This retrospective chart review study has been conducted by reviewing the records of Tuberculosis Management Unit in Assiut Chest Hospital. Assiut Chest Hospital was built in 1943 to fight tuberculosis in Upper Egypt. Nowadays, the Tuberculosis Management Unit occupies the old building while the new ones serve other chest diseases. The most recently constructed building, including the emergency room (ER) and the respiratory intensive care unit (RICU), was transferred to COVID-19 isolation hospital by the beginning of the pandemic. The tuberculosis management unit collects data of 12 healthcare centers with TB patient management programs all over Assiut Governorate, Egypt. GenXpert (Xpert MTB/RIF assay) was first introduced there in 2015. A total of 2400 samples have been diagnosed with TB and rifampicin resistance.

For the current study, the following data were obtained through the registration system from 2017 to the first two quarters of 2021: new smear-positive and smear-negative cases, retreated cases, treatment failure, defaulters, and new extrapulmonary cases.

For each quarter, the incidence rates (IR) were calculated. Pulmonary TB was estimated by dividing new and relapsed pulmonary cases over 100,000 population. Moreover, extrapulmonary TB was measured by dividing new and relapsed extrapulmonary cases over 100,000 population. The overall IR for both pulmonary and extrapulmonary cases was also calculated. Comparison between incidence rates was done using Medcalc software based on chi square statistics and *P* value < 0.05 considered as significant. Besides, the yearly incidence was calculated from 2017 to 2020. For incidence rate calculations and plotting of line graphs, MS Excel was used. The quarterly percentage change in TB cases numbers was calculated by dividing the difference between new and old cases by the old cases multiplied by 100, (2020 relative to 2019 and 2021 to 2019).

$$\mathrm{Percentage}\ \mathrm{change}=\frac{\mathrm{New}\ \mathrm{cases}-\mathrm{Old}\ \mathrm{cases}}{\mathrm{Old}\ \mathrm{cases}}\times 100$$

The study was approved by the institutional review board and ethical committee in compliance with the Helsinki Declaration (IRB: 17300480).

## Results

**Fig. 1:**
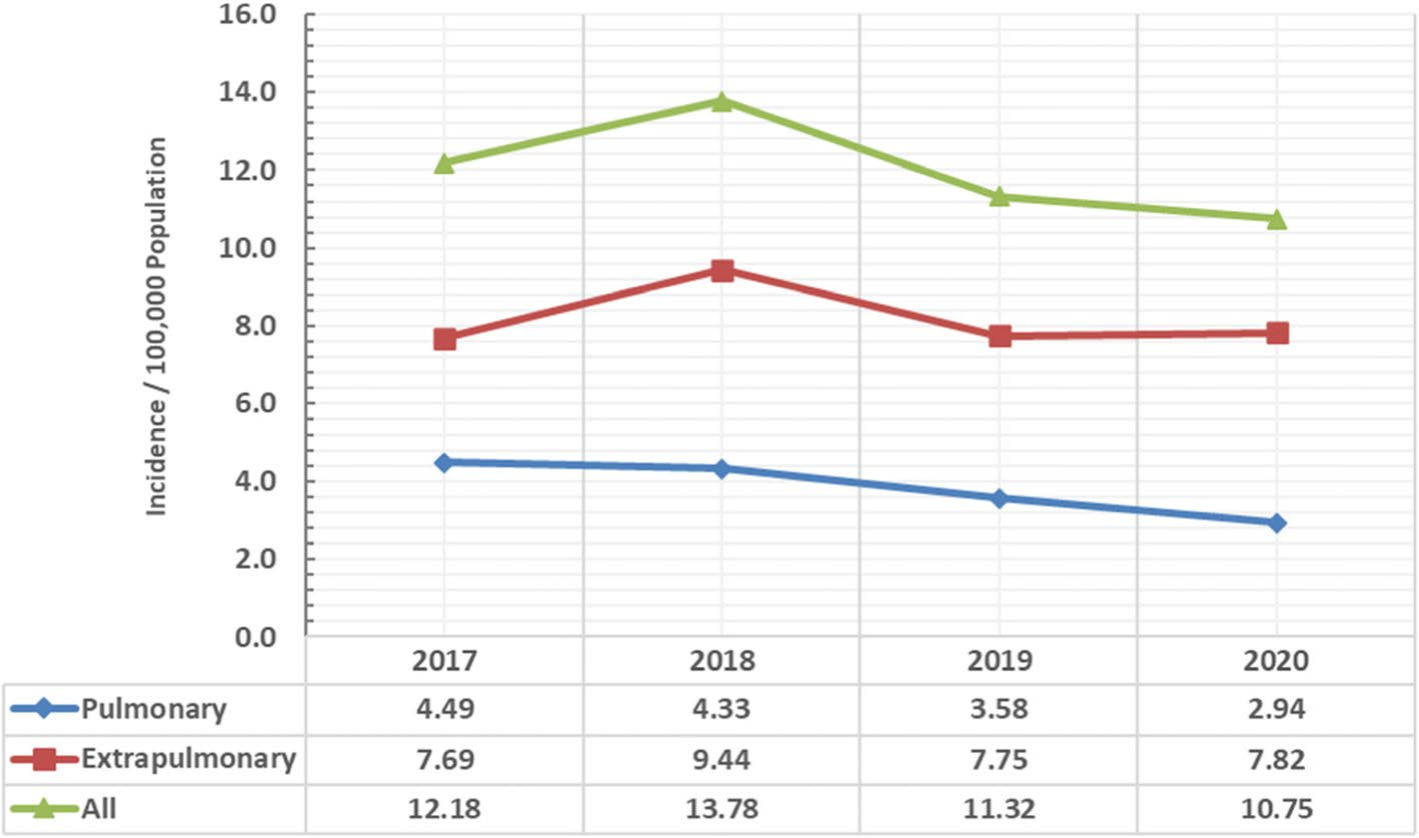
Yearly TB incidence from 2017 -2020 in Assiut Chest Hospital, Egypt

A total 2256 TB cases were diagnosed in Assiut Chest Hospital from 2017 to 2020 and 237 cases in the first 6 months of 2021. Extrapulmonary cases were more than pulmonary during the study period. There was a gradual decline in the incidence of TB from 12.18 case/100,000 population in 2017 to 10.75 case/100,000 population in 2020 and this difference is statistically significant (*p* value = 0.044) (Fig. 1).

**Fig. 2:**
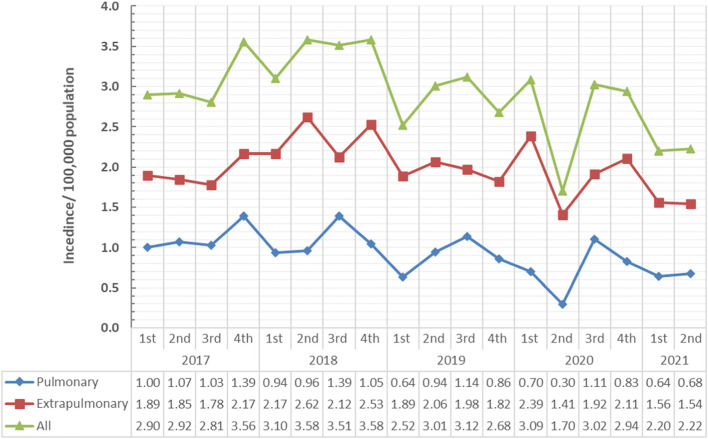
TB incidence over quarters of years (2017–2021) in Assiut Chest Hospital, Egypt

**Fig. 3:**
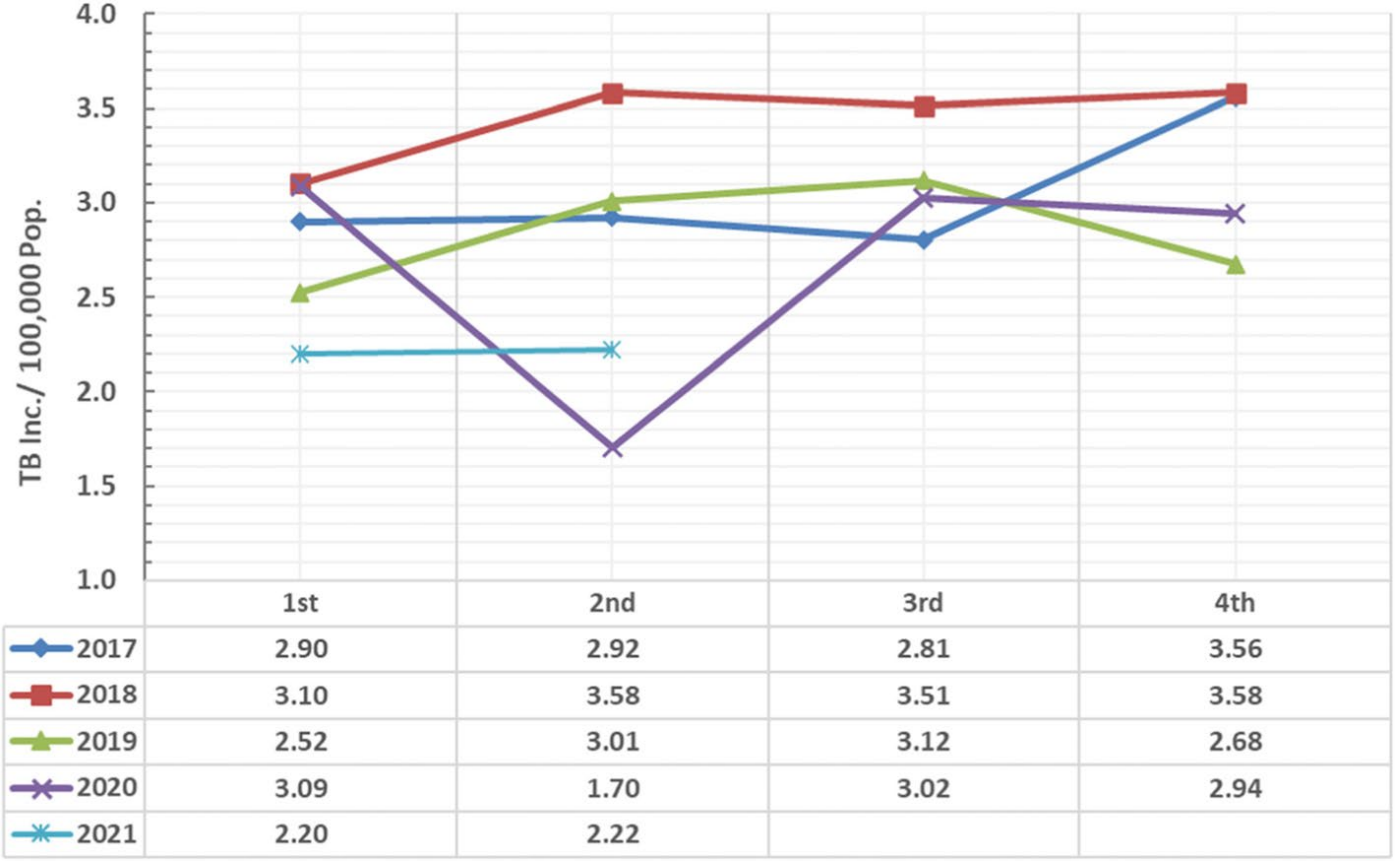
TB incidence trends in different years (2017–2021) with comparison between quarters in Assiut Chest Hospital, Egypt

The TB case notification has been markedly declined at the second quarter of 2020 (incidence rate difference for 2020 1st quarter vs. 2nd quarter = − 1.39/100,000 population, *p* value < 0.001) and then rose to its previous levels, then steadily decreased in the first and second quarters in 2021. These patterns of change were observed in all TB cases, either pulmonary or extrapulmonary (Fig. 2). Figure 3 demonstrated the incidence trend of all TB cases, pulmonary and extrapulmonary, in different quarters of years 2017–2021. As illustrated, the second quarter of 2020 showed a deep decline in all cases. Incidence rate difference of different years is presented in Table 1.

**Table 1:** Tuberculosis incidence rate difference in Assiut Chest Hospital for different years (2017–2020)

Years	Incidence rate difference (Per 100,000)	***p*** value*
2017 vs. 2018	− 1.6	0.038^
2017 vs. 2019	0.86	0.236
2017 vs. 2020	1.43	0.044^
2018 vs.2019	2.46	0.001^
2018 vs. 2020	3.03	< 0.001^
2019 vs. 2020	0.57	0.410

*Chi-square test

^Significant *p* value

**Fig 4:**
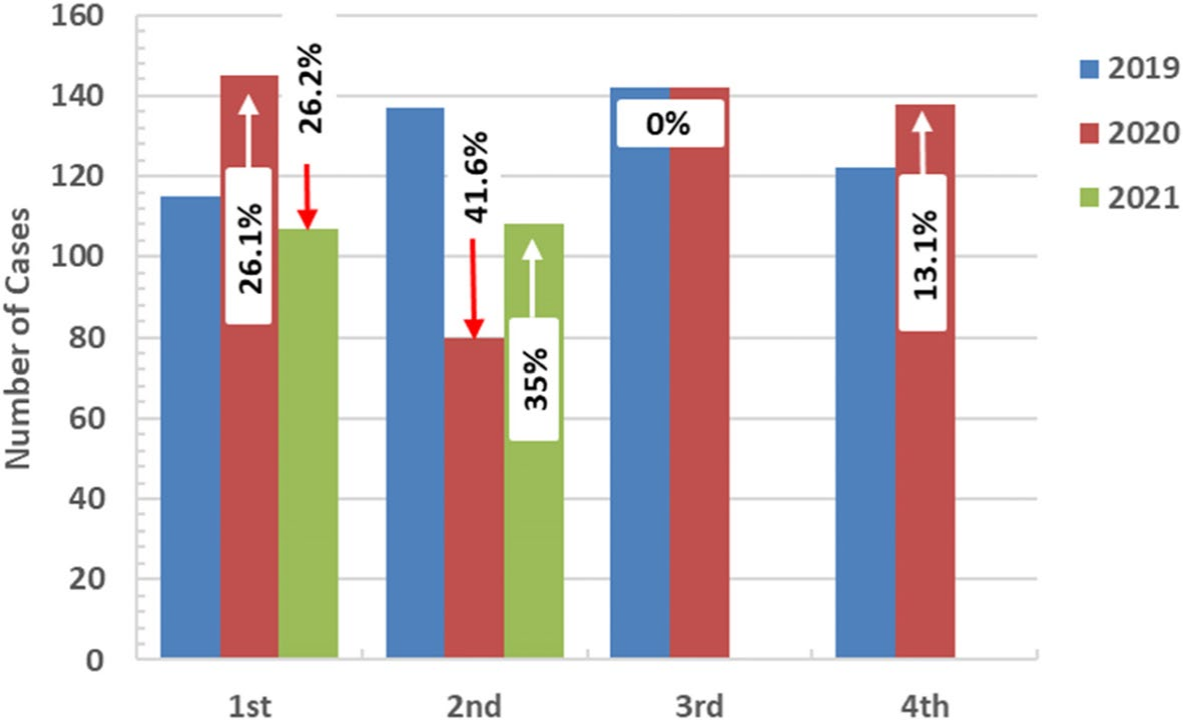
Percent changes in number of TB cases in different quarters (2020 vs. 2019) and (2021 vs. 2020) in Assiut chest hospital, Egypt. (↑=increase, ↓=decrease)

**Fig. 5:**
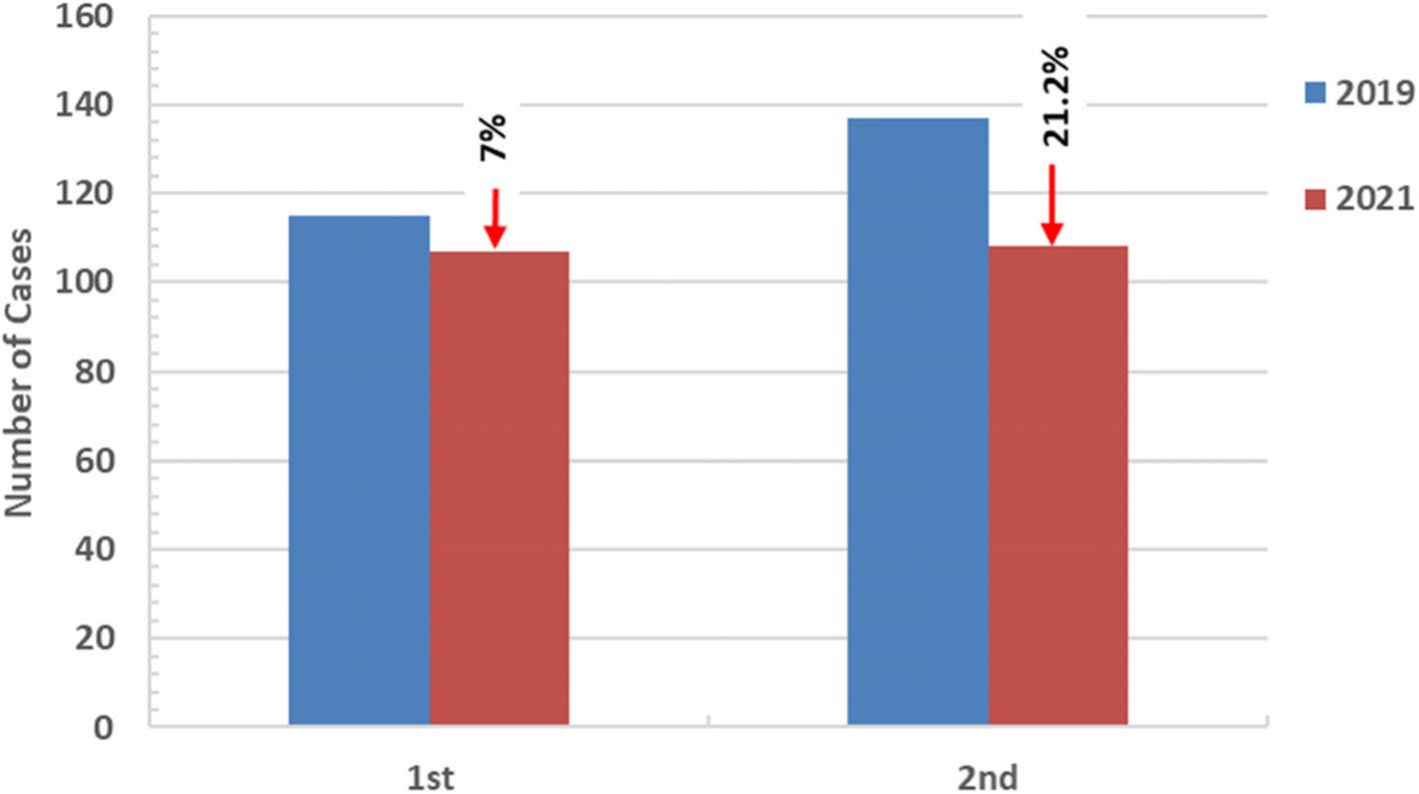
Percent changes in number of TB cases in first two quarters (2021 vs. 2019) in Assiut Chest Hospital, Egypt. (↓ = decrease)

The percentage of changes in the numbers of reported cases has been demonstrated in Figs. 4 and 5. Comparing 2020 with 2019 showed an increase in the first quarter by 26% and a decrease by 41.6% in the second quarter (with 67.4%, 29.8% decline in the number of the diagnosed cases of pulmonary and extrapulmonary tuberculosis, respectively), was reported. The third quarter showed no changes. In the last quarter, the cases increased by 13%. Comparing the first two quarters of 2021 with 2020 demonstrated a first-quarter decline of 26% and a second-quarter increase of 35%. Moreover, comparing the first two quarters of 2021 to the same period in 2019 demonstrated a decrease of 7%, 21%, respectively. Four cases were diagnosed as multidrug-resistant tuberculosis (MDR-TB) in 2020, and only one case in the first two quarters of 2021. Since 2015, 42 cases have been diagnosed as MDR-TB, with an average of eight cases yearly (ranging from 7 to 11 cases per year in the pre-COVID-19 era). Moreover, in 2019, eight cases of MDR-TB were diagnosed with 50% drop in MDR-TB detection during the pandemic. However, this was not included in the statistical analysis due to the small number.

## Discussion

Since being declared a global pandemic, COVID-19 exhibits a great burden on healthcare services. To evaluate the impact of COVID-19 on tuberculosis case detection, this retrospective chart review study has been conducted. By the beginning of total lockdown in Egypt due to COVID-19 pandemic, a noticeable decline in the incidence of pulmonary TB during 2020 was recorded. The second quarter of 2020 showed the lowest tuberculosis incidence rate with a 41.6% decline in the diagnosed cases. A 67.4% and 29.8% decline in pulmonary and extrapulmonary tuberculosis cases were reported, respectively.

Tuberculosis is considered one of the most critical healthcare problems in Egypt. The incidence of tuberculosis in Egypt decreased gradually from 26 cases per 100,000 people in 2000 to 12 cases per 100,000 people in 2019 [3]. In the current study, tuberculosis decreased from 12.18 cases per 100,000 people in 2017 to 10.75 cases per 100,000 people in 2020. Nevertheless, extrapulmonary tuberculosis was higher than pulmonary tuberculosis throughout the 4 years reviewed in the study. These findings are inconsistent with data showing that extrapulmonary TB was more prevalent by 64.14% in a previous study performed in the exact center [12], and 51% in another center in North Africa [13]. Risk factors for extrapulmonary TB include higher age, female gender, geographical distribution, birth in high TB-prevalent countries, exposure at place of residence or work, homelessness, and presence of other comorbid conditions [14, 15]. Moreover, the COVID-19 pandemic witnessed a noticeable decline in TB incidence, especially in the second quarter of 2020 and the first and second quarters of 2021, correlating with many TB management settings reports [16–19].

The COVID-19 pandemic overwhelmed the healthcare system worldwide, including TB screening and management facilities. Three months after the pandemic, an investigation was carried in 165 countries, with 42% reporting partial interruptions in tuberculosis case detection and treatment [20]. After finalizing data for 2020, a 21% fall in tuberculosis notifications was recorded globally from 2019 to 2020. A substantially more significant reduction was demonstrated in nations with high burdens of TB, such as Indonesia, India, South Africa, and the Philippines. Substantial interruptions will continue in several countries, as indicated by the Primary information for 2021. Countries with a high prevalence of tuberculosis, such as Indonesia, Angola, Myanmar, and Lesotho, witnessed a decline of more than 25% compared to the average for 2019 [21]. In the current study, there was a marked decline in TB incidence during the second quarter of 2020 by a 41.6% decline in the total number of diagnosed cases; 67.4% and 29.8% decline in cases of pulmonary and extrapulmonary tuberculosis, respectively. However, the following quarters of 2020 showed restoration of the incidence rate toward that of the same period in the previous year. It may be assumed that the drop in the second quarter was related to the lockdown by the end of March 2020, thus resulting in limited healthcare resources following the national TB program and a reduction in public transportation hours that made the access to public health services more difficult.

Moreover, the growing fear of getting COVID-19 infection during the pandemic’s peak resulted in missed or delayed diagnosis of TB cases. These assumptions were supported by reports of COVID-19 prevention assessments requiring the use of facemasks and social distancing, reduced public attendance at healthcare facilities due to fear of infection with COVID-19, reduced healthcare worker capacity due to the closure of numerous tuberculosis outpatient clinics, a lack of personal protective equipment in some health facilities forcing them to cease conducting tuberculosis tests temporarily [16, 22].

The rise in the third quarter incidence might be due to the cumulative cases seeking medical services after the lockdown period has been ended. Another hypothesized cause for that rise was the increased indoor/household infections. Again, the first two quarters of 2021 showed a steady drop in both pulmonary and extrapulmonary TB incidence despite the end of lockdown and restoration of most daily activities with the protective measures being applied. The possible explanations for this phenomenon were: the fear of the TB patients from getting COVID-19 infections during transportation to the healthcare facility or from healthcare workers themselves, especially after using Assiut Chest Hospital main building as a COVID-19 isolation hospital. The similarities in symptoms between TB and COVID-19 masked some TB cases who were treated empirically as COVID-19 or refused to seek medical advice. The exhaustion of human resources and equipment in health care facilities affected the competency and efficacy of tuberculosis screening programs and the direction of media and social awareness activities, and programs towards COVID-19 prevention are all assumed causes for this continues decline.

The continued decline in TB case detection during 2021 clarify that the burden of COVID-19 on TB is not a temporary problem. With COVID-19 continue to grow and spread, there is an urgent need to restore TB case detection and treatment efficacy to pre-COVID-19 levels. Numerous suggestions to improve TB services in the face of the pandemic have been studied, with being a financial issue and a state of knowledge and awareness. First, improved integration of COVID-19 diagnosis and tuberculosis screening at the community and health facilities is required. Second, laboratories must share testing methodologies and multiplexing equipment like GeneXpert platforms. Third, Assuring effective infection, control activities, and prevention within health facilities. Fourth, mobilizing TB networks supporting communities and survivors. Fifth, providing extended 3-month follow-up consultations for drug collection and patient check-ups. Sixth, increasing digital platforms used for drug adherence, case finding, and management. Seventh, spreading awareness in communities and health facilities. It was also advised that recently developed geospatial tracking systems be repurposed to identify contacts of tuberculosis and that virtual systems be used to verify compliance with treatment. Additionally, the extraordinary resources and cash gathered to tackle the pandemic should be used to also tackle TB, with a priority to the poor [17, 23–27]. In Egypt, application of the WHO control strategy for TB, including Bacillus Calmette-Gue´rin (BCG) vaccine at birth, case detection, and treatment of cases with directly observed therapy short-course (DOTS), is mandatory even during the pandemic. More attention is needed to improve case detection by increasing the awareness and diagnostic facilities in the primary healthcare centers making them closer and more accessible to the patients.

Limited information about TB cases and their demographic and social characteristics resulted in difficulty to track the nature of care for TB cases during COVID-19. Thus, the efficacy of the diagnosis system concerning local circumference was not evaluated in depth in this study. Moreover, the commitment to treatment and visits rates and regularity to outpatient clinics, by patients diagnosed to have TB either before or during the pandemic, was not evaluated. Thus, the infection sequelae and treatment outcome could not be known accurately. Finally, the impact of the COVID-19 pandemic on MDR-TB is needed to be studied nationally, considering a greater sample size.

## Conclusion

There was a noticeable drop in tuberculosis case detection and follow-up during the COVID-19 pandemic. The lockdown led to a remarkable decline in the second quarter of 2020. However, a steady partial decline was continued during the first and second quarters of 2021, demonstrating a growing problem. Concerns must be raised against the burden of the pandemic on tuberculosis diagnosis and management programs to avoid affecting TB patients' prognosis and survival.

## Data Availability

The datasets used and/or analyzed during the current study are available from the corresponding author on reasonable request.
